# Isolation and Genomic Characteristics of a Novel Pathogenicity Type I Feline Coronavirus in Mainland China

**DOI:** 10.1155/2024/4162458

**Published:** 2024-11-13

**Authors:** Yuanhong Wang, Junna Wang, You Zhao, Yun Liu, Miao Zhang, Xiaoying Deng, Jie Zhu, Guoxin Li, Guangqing Liu

**Affiliations:** ^1^Shanghai Veterinary Research Institute, Chinese Academy of Agricultural Sciences, Shanghai 200241, China; ^2^Research Center of General Administration of Customs, Beijing 100011, China

**Keywords:** feline coronavirus (FCoV), isolation, pathogenicity, recombination

## Abstract

Feline coronavirus (FCoV) is an enveloped, positive-sense RNA virus, which is widespread among feline populations, and can cause a fatal serious disease called feline infectious peritonitis (FIP). According to the differences of antigen and genetic composition, FCoV consists of two genotypes, FCoV I and FCoV II. In this study, we have isolated and identified a FCoV I strain named HL2019. Based on the complete genome of HL2019, phylogenetic analysis showed that HL2019 strain formed in the cluster FCoV I which is more closed to human coronavirus 229E (HCoV 229E) and HCoV NL63, while the FCoV I stains is distantly related to FCoV II strains. Analyzing with RDP4 and Simplot software showed that the virus HL2019 is recombinant by the FCoV I China/ZJU1709 and FCoV I Netherlands/UU16 strains. Furthermore, the pathogenicity of HL2019 was evaluated in 9–12-month-old cats. Two of three challenged cats developed serious clinical signs and died at 28-day postchallenge (dpc). Real-time polymerase chain reaction (PCR) analysis showed that HL2019 has broad tissue tropism, especially in the duodenum with viral load up to 10^4^ copies/mg. In summary, our data show that we have successfully isolated a strain of FCoV I named HL2019 that is highly pathogenic to cats.

## 1. Introduction

Feline coronavirus (FCoV) is an enveloped, positive-sense RNA virus, which belongs to the genus *Alphacoronavirus* of the subfamily *Orthocoronavirinae*, family *Coronaviridae* [1]. FCoV infection is widespread among feline populations, especially within multicat households and catteries, which can exhibit upward of 96% seropositivity [2, 3]. Based on biological characteristics, FCoV is typically divided into feline enteric coronavirus (FECV) and feline infectious peritonitis virus (FIPV). Most cats with FECV do not show any or only mild symptoms, such as diarrhea and anorexia [4]. But FIPV could cause a more serious disease called feline infectious peritonitis (FIP), which results in fatal disease in cats once clinical signs develop [5].

As companion animal, people are increasingly concerned about the physical health of cats. Previous studies have shown that severe acute respiratory syndrome coronavirus 2 (SARS-CoV-2) can replicate in the cats' nose and throat and caused inflammatory pathology deeper in the respiratory tract, and airborne transmission did occur between pairs of cats [6]. In addition, a Malaysian study has identified a novel canine–feline recombinant alphacoronavirus named CCoV-HuPn-2018 which can infect human [7]. This suggests the possibility of cross-species transmission of CoV between human and feline. Tracking the origin of emerging pathogens is crucial for controlling their spread, and research on the transmission of pathogens with different species helps prevent pandemics [8].Therefore, it is necessary to figure out the epidemiology and biological characteristics of FCoV.

In this study, we first isolated a FCoV I strain named HL2019 in mainland China. We provide molecular characterization and pathogenicity of the virus HL2019. The results showed that this virus was highly pathogenic in cats and replicated efficiently in the duodenum. And this study will be helpful for understanding the evolution and pathogenicity of FCoV in mainland China.

## 2. Materials and Methods

### 2.1. Ethics Statements

We performed the animal experiment following the guidelines of the Institute of Shanghai Veterinary Research, Chinese Academy Agriculture of Sciences, using protocols approved by the Institutional Laboratory Animal Care and Use Committee (SV-20230728-01).

### 2.2. Sample Collection

Clinical samples (cat spleen and ascites) were collected from the British shorthair cat in Anhui province, China, in 2019. The cat showed a typical FIP signalment, including effusion, hyperproteinemia, hyperglobulinemia, hypoalbuminemia, and hyperbilirubinemia.

### 2.3. Isolation and Identification of HL2019 Strain

To isolate FCoV, we aseptically treated the effusion and inoculated it into a monolayer of FCWF-4 cells (American Type Culture Collection (ATCC): B184268), which were growing in Eagle's Minimum Essential Medium (EMEM; Hyclone, USA) supplemented with 5 μg/mL 0.25% (w/v) trypsin (Gibco, Australia) in 5% CO_2_ at 37°C. After ~80%–90% of the cells exhibited cytopathic effect (CPE), we conducted repeated freezing and thawing, and then we eliminated cell debris by centrifugation at 1500 × *g* for 5 min at room temperature. The supernatant containing the mature viral particles was stained with 2% phosphotungstic acid and observed with electron microscope (EM).

### 2.4. Western Blot and Immunofluorescence Assays

After 24 h postinfection, we fixed FCWF-4 cells infected with the virus HL2019 in 6-well plates at a one multiplicity of infection (MOI) with 4% paraformaldehyde. Subsequently, we used the FCoV I N-protein polyclonal antibody (prepared in our laboratory) as the primary antibody (1:100), followed by fluorescent isothiocyanate (FITC)-labeled goat antimouse secondary antibody (1:10,000) (Beijing, ZSBio) to examine fluorescence. We counterstained with 4′,6-diamidino-2-phenylindole (DAPI) at room temperature for 5 min and used a fluorescence microscope (Nikon, Tokyo, Japan) for examination. And we also performed blank FCWF-4 cells in immunofluorescence assay (IFA) as a negative control. In addition, the primary antibody FCoV I N-protein polyclonal antibody [9] and the secondary antibody horseradish peroxidase (HRP)-conjugated goat antimouse immunoglobulin G (IgG; Beijing, ZSBio; 1:5000) were subjected to the western blot assay.

### 2.5. Next-Generation Sequencing (NGS)

The HL2019 P3 was harvested for the extraction of viral RNA. Illumina sequencing and library construction were performed at the Shanghai Tanpu Biotechnology Co., Ltd (Shanghai, China). Specifically, the NEBNext Ultra II RNA Library Prep Kit (NEB, Ipswich, MA, USA) was used for library construction. After adapter ligation, 10 cycles of polymerase chain reaction (PCR) amplification were used to enrich the sequencing target. The libraries were pooled in equimolar amounts, denatured, and diluted to the optimal concentration before sequencing. The Illumina NovaSeq 6000 System (Illumina, San Diego, CA, USA) was used for sequencing to generate 150-bp pair-end reads.

### 2.6. Sequence Datasets

The sequences we have used in this study were obtained from the NCBI GenBank database (https://www.ncbi.nlm.nih.gov/), and 73 FCoV strains were downloaded (accessed on June 31, 2023). Specifically, 24 strains of canine coronavirus (CCoV), 46 strains of transmissible gastroenteritis virus (TGEV), 5 strains of human coronavirus (HCoV) NL63, 5 strains of HCoV 229E, and 5 strains of bat alphacoronavirus (BatCoV) were used to understand the relationship between *Alphacoronavirus*. To be specific, we downloaded five strains of SARS-CoV-2 which are from *Betacoronavirus* (Table 1).

### 2.7. Phylogenetic Analysis

For comparing and sorting the complete genome, ModelFinder software was used to select best model which is generalized time-reversible (GTR)+F+G4 and assume an uncorrected relaxation clock (lognormal) [10, 11]. The chain length for each calculation is set to 5 × 10^9^ generations, obtained every 50,000 generations, with a total of three independent operation sets. After burn-in (10%), the Tracer software (V1.7.1) was used to estimate the data. Parameters with an effective sampling size of more than 200 were accepted [12]. FigTree software (V1.4.4) was using to display the final maximum clade credibility (MCC) tree.

### 2.8. Recombination Screening

For understanding the potential recombination events, the occurrence of potential recombination events between complete genome sequences of 163 strains in this study was analyzed using seven algorithms of the RDP4 software package, including RDP, GENECONV, Bootscan, MaxChi, Chimaera, SiScan, and 3Seq [13]. The recombination event identified by at least four of the seven methods was accepted in this report; the recombination events were confirmed by at least four methods with a *p*-value cutoff of 0.05. Furthermore, the SimPlot 3.5.1 software was used to confirm the potential recombinant events occurred in HL2019 by using the neighbor-joining method in the Kimura 2-parameter (K2P) model [14]. The window size and step were set to 200 bp and 20 bp, respectively, with a *p*-value cutoff of 0.05.

### 2.9. Animal Experiments

In this study, for the cats used in this experiment, we have used reverse transcription PCR (RT-PCR) to confirm feline calicivirus (FCV) and FCoV were pathogen-free and PCR to confirm feline panleukopenia virus (FPV) and feline herpesvirus (FHV) were pathogen-free (Table S1). Then the six 9–12-month-old cats were randomly divided into two groups: HL2019 group (*n* = 3) and Mock group (*n* = 3). All animals were housed in the P2 laboratory. Cats in HL2019 group were inoculated orally with isolated virus (10^5^ TCID_50_/cat), while cats in the Mock group were inoculated orally with phosphate-buffered saline (PBS). According to Thayer et al. [15] which have published the Diagnosis Guidelines in 2022, the clinical signs and symptoms were monitored daily. Two of the cats inoculated with the virus died after 3 weeks. The copy numbers of the heart, liver, spleen, lung, kidney, duodenum, and ascites were obtained by *N* EvaGreen real-time RT-PCR [5]. The duodenum was also analyzed for hematoxylin and eosin (H&E) and immunohistochemistry (IHC) stain.

## 3. Results

### 3.1. Sequencing and Virus Isolation

**Figure 1:**
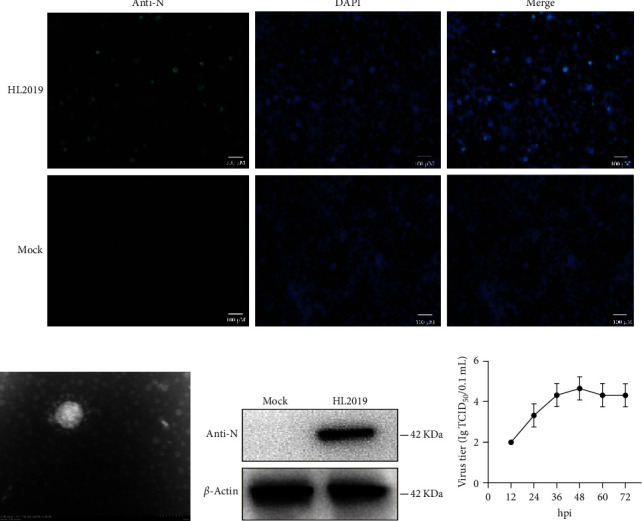
Characteristics of isolated FCoV: (A) immunofluorescence assay identification of HL2019 strain; (B) transmission electron microscope observation of HL2019; (C) western blot identification of HL2019 strain; (D) multistep replication curves of HL2019 in FCWF-4 cells at 12, 24, 36, 48, 60, and 72 hpi. Newly isolated viruses HL2019 were used to infect FCWF-4 cells at a MOI of 1. DAPI, 4′,6-diamidino-2-phenylindole; FCoV, feline coronavirus; MOI, multiplicity of infection.

After 72 h, FCWF-4 cells exhibited cytopathic effects, including shrinking, rounding, lighting, and disruptive morphological characteristics (Figure 1A). And the typical crown particle was observed under transmission electron microscopy (TEM) with a diameter of about 100 nm (Figure 1B). Meanwhile, western blot analysis results indicated that the isolated virus (named HL2019) could specifically react with FCoV I N-protein polyclonal antibody (Figure 1C). Multistep replication curves revealed that the mean virus titer of HL2019 was the highest at 48 hpi (Figure 1D).

Furthermore, we have sequenced the complete genome of HL2019 P3; the results showed that the complete nucleotide sequence of HL2019 consisted of 29,044 nt (excluding the poly [A] tail) and contained 11 open reading frames (ORFs), which encoded four structural proteins (S, E, M, and N), five accessory proteins (7a and 7b, 3a, 3b, and 3c), and two nonstructural proteins (1a and 1b). The poly (A) tail of FIPV had 20 A's at least on either side of the coding region of the genome are 5′ untranslated region (5′UTR) and 3′UTR, which are 294 nt and 247 nt long, respectively.

Our data confirmed that we have successfully isolated a FCoV I HL2019 strain. The genomic sequence of HL2019 has been submitted to GenBank, and its accession number is OR475582.

### 3.2. Potential Recombination Events in HL2019 Strain

**Figure 2:**
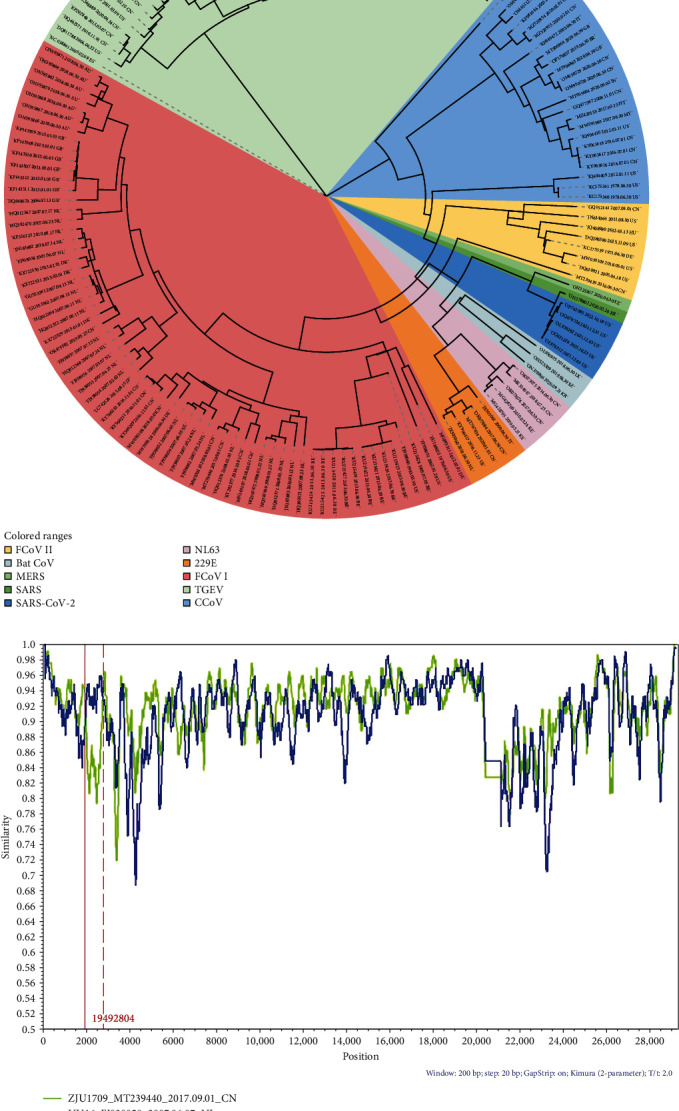
The phylogenetic and recombinant analysis of FCoV. (A) The phylogenetic analysis of FCoV was deduced using the complete genome of the different coronaviruses. MCC tree was reconstructed using BEAST (1.10.4). The different coronaviruses are expressed by different RGB colors as indicated. HL2019 is labeled in green. (B) The recombinational event was evaluated in Simplot for the complete genome of MT239440 (FCoV ZJU1709), FJ938058 (FCoV UU16), and OR475582 (FCoV HL2019). BatCoV, bat alphacoronavirus; CCoV, canine coronavirus; FCoV, feline coronavirus; MCC, maximum clade credibility; MERS, Middle East respiratory syndrome; RGB, red, green, blue; SARS, severe acute respiratory syndrome; SARS-Cov-2, severe acute respiratory syndrome coronavirus 2; TGEV, transmissible gastroenteritis virus.

In this study, RDP4 was used to detect recombination and confirmed that there has recombinational event that existed among strains; we found that MT239440 (FCoV I ZJU1709) and FJ938058 (FCoV I UU16) might represent the parent lineages of OR475582 (FCoV I HL2019). Similarity plot and bootscanning analyses predicted the potential breakpoints in HL2019 to be at ORF1a(nt 2021 and 2821) (Figure 2A). ORF1a encodes a papain-like protease (PLpro) which is essential for coronaviral replication [16]. The occurrence of recombination and mutation events in ORF1a region may be a crucial strategy employed by FCoV to evade innate and adaptive immunity.

### 3.3. Relationship Among Multiple Species of Coronavirus

In order to analyze the phylogenetic relationship between different species of coronavirus, we download some alphacoronavirus complete genomes for reconstructing MCC (Figure 2B). The results of the phylogenetic trees showed the HL2019 strains located in the clade of FCoV I and closed to the UG-FH8 which is isolated from Denmark. The clade of FCoV I stains was distantly related to clade of FCoV II strains, while the clade of FCoV II strains was closed to TGEV and CCoV strains. Interestingly, we found that the clade of FCoV I showed high confidence with HCoV NL63 and HCoV 229E. Further analyzed on the homology of HCoV NL63 (MG428705.1), HCoV 229E (JX503060.1), and FCoV I HL2019 (OR475582), it is showed that the homology of HCoV NL63 (MG428705.1) and HL2019 (OR475582) is up to 60.1%, and the homology of HCoV 229E (JX503060.1) and HL2019 (OR475582) reached to 61.2%. It indicated that the FCoV may have cross-species transmission potential.

### 3.4. HL2019 Is Highly Pathogenic to Cats

**Figure 3:**
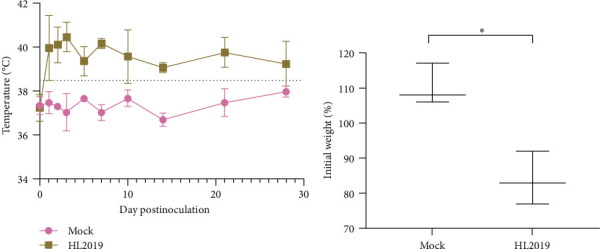
Pathogenicity evaluation of HL2019 strain. (A) The cat ear temperatures responded to viral infection over time. (B) Average relative weight at 28-day postchallenge (dpc). On 0 dpc, the body weight was 100% *⁣*^*∗*^*P* < 0.5.

**Figure 4:**
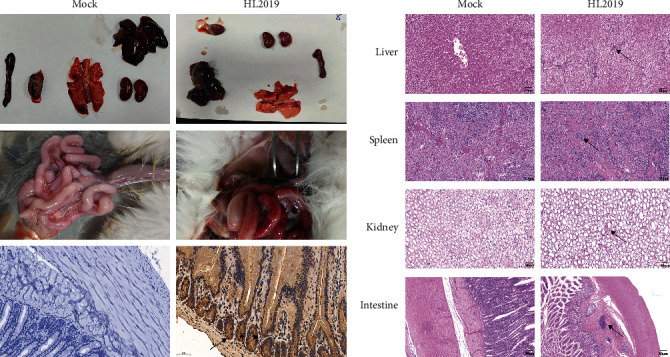
Necropsy and histopathology analysis. (A) Gross necropsy of the cat euthanized with effusive FIP and IHC-stained sections of intestine sections. Effusion was observed in the abdomen at necropsy. In the IHC images, viral antigen was detected in the FIP lesions of the intestine by antimouse FCoV N protein antibody (brown). (B) H&E-stained sections of the liver, speen, kidney, and intestine sections. Typical FIP perivasculitis contained B-cell and plasma cell infiltrates in leptomeninges, and distinct focal accumulations of macrophages were observed just under the capsule of the liver, speen, kidney, and intestine. These foci were surrounded by dense accumulations of lymphocytes and plasma cells, extending downward into the parenchyma. While mocks remain normal. FIP, feline infectious peritonitis; H&E, hematoxylin and eosin; IHC, immunohistochemistry.

Prior to the challenge, all cats were healthy. After oral challenge with HL2019 (10^5^ TCID_50_/cat), two of the three cats showed typical sign associated with FIP including inappetence, anorexia, weight loss, fever, and diarrhea and died within 28-day postchallenge (dpc). Specifically, cats in the HL2019 group developed a persistent fever after virus challenge, while cats in the Mock group maintained normal (Figure 3A). Additionally, the average weight of cats in the HL2019 group (84% of initial weight) was significantly lower than the Mock group (110% of initial weight) (Figure 3B). On 28 dpc, all cats were euthanized. In the HL2019 group, the ascites were observed, with an intestinal bleeding and congestion (1/3), while the Mock group remain normal, and the IHC results further confirmed that viral antigen was detected within macrophages in the lesions of cats from the HL2019 groups (Figure 4A).

For H&E examination, distinct focal accumulations of macrophages were observed just under the capsule of the liver, spleen, kidney, and intestine in the HL2019 groups, while the Mock groups remain normal (Figure 4B).

### 3.5. Detection of Virus Load

**Figure 5:**
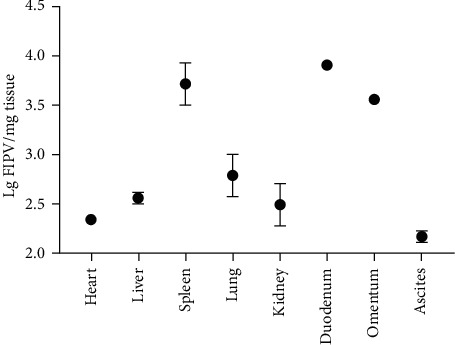
Detection of the virus load in different tissues.

FCoV antigens were detected in organs, such as the lung, heart, spleen, liver, kidney, duodenum, omentum, and ascites. And it is abundant in the duodenum and spleen which is reaching 10^4^ copies/mg (Figure 5).

## 4. Discussion

FCoV processes about 29 k-nucleotide positive-sense single-stranded genomic RNA, which is composed of 11 ORFs encoding 7 nonstructural proteins (1a, 1b, 3a, 3b, 3c, 7a, and 7b) and 4 structural proteins (the spike, envelope, membrane, and nucleocapsid proteins) [2, 3]. According the spike sequence, FCoV has divided into two kinds of serum type (FCoV I and FCoV II) [17, 18]. FCoV I accounts for the bulk (80%–90%) of natural infections in cats, while FCoV II (naturally occurring recombination between FCoV I and CCoV spike proteins) is far less prevalent (<10%) [19]. FCoV I is difficult to be cultured in vitro, while FCoV II is easier to grow in cell cultures. Previous studies have reported FCoV I to be isolated, but the titer has not been defined [20, 21]. In our study, we have successfully isolated a FCoV I strain on FCWF-4 cells, and its titer is reaching 10^5^ TCID_50_ which is important to study the pathogenic mechanism of FCoV I and development of related biological products against FCoV.

Recombinant analysis was performed to examine the evolution of the isolate HL2019 strain. The HL2019 is located in cluster FCoV I and most closely related to UG-FH8 which was isolated in Denmark. In addition, recombination analysis indicated that a potential recombination event had occurred within ORF1a. The primary function of PLpro is to process the viral polyprotein in a coordinated manner. PLpro has the additional function of stripping ubiquitin and ISG15 from the host cell proteins to aid coronaviruses in their evasion of the host innate immune responses [22, 23]. Of note, increasing studies point to that cats can be the intermediate hosts for some HCoVs, such as SARS-CoV-2 and HCoV-229E [24, 25]. In addition, feline-like CoV strains have been reported to infect humans [26], suggesting that FCoV has the potential for interspecies transmission or evolve as a new human strain. In this study, we have found that the FCoV I strains are more closed to HCoV, including HCoV 229E and HCoV NL63, suggesting cats play a potential role in interspecies transmission of coronaviruses. So it is necessary to conduct more in-depth research to dig out the viral variability and evolution of FCoV.

Furthermore, our results showed that this virus was pathogenic in 9–12-month-old cats. Meanwhile, real-time PCR analysis showed that HL2019 has broad tissue tropism; FCoV was abundant in the duodenum which is up to 10^4^ copies/mg. In addition, the levels of viral genomic RNA paralleled the intensity of the inflammatory response, which in turn paralleled the numbers of infected macrophages in the tissues or effusion. Accoring to Kiss, Poland, and Pedersen [27] study, they used an avirulent type I strain of FIPV-UCD1 and challenge exposed to a highly virulent cat passaged type I strain FIPV-UCD8. They found a FIPV-UCD1 immunization induced only partial protection at best, as gauged against historical data. Animal-passaged FIPV-UCD8 usually kills from 90 to 100% of inoculated cats, almost always from effusive FIP. And in Terada et al. [28] study, they discovered C3663, a strain of FCoV I isolated from FIP cats that retained virulence despite adaptation in FCWF-4 cells. It could make three (75%) of four SPF cats developed FIP after infection with the C3663 strain, and in their further research, they found the cDNA clone for type I FCoV strain C3663. It seems the virulence of FCoV I strains varies greatly, and the specific mechanism still needs further study.

## 5. Conclusion

In a word, this is the first study to report the complete viral genome of FCoV I strain HL2019 isolated from FCWF-4 cells in the mainland of China. We provide evidence of the pathogenicity of the virus HL2019 which provided scientific evidence that HL2019 could be highly pathogenic to cats. These results provide important information on the evolution of FCoV in mainland of China and suggest that we need to continue surveillance studies in felines to monitor the spread and evolution patterns of FCoV and other CoV infections, as well as the detection of recombinant viruses.

## Figures and Tables

**Table 1:** CoVs reference strains used in this study.

GenBank ID	Collection time	Country	Strain
KX722530.1	1-Feb-15	Denmark	Cat 1 Karlslunde
KX722529.1	1-Jan-15	Denmark	UG-FH8
MW308128.1	2006	Germany	VP1a
MG893511.1	Jul-12	Germany	Felix
KX722531.1	1-Feb-15	Denmark	Cat 2 Holstebro
KY566211	Nov-16	China	HLJ/HRB/2016/13
KY566210	Nov-16	China	HLJ/HRB/2016/11
KY566209	Nov-16	China	HLJ/HRB/2016/10
KY292377	Oct-16	China	HLJ/DQ/2016/01
GQ152141	Sep-07	Taiwan	FCoV/NTU156/P/2007
DQ848678	13-Jul-06	United Kingdom	FCoV C1Je
DQ010921	18-Apr-05	United States	FIPV 79-1146
EU186072	1970	United States	Black
DQ286389	9-Nov-05	United States	DF-2
LC742526	2015-Aug-15	Japan	FCoV/I/JP15/Fe/35/2015
ON595871	2018	Australia	HC-10
ON595870	2018	Australia	HC-9
ON595869	2018	Australia	HC-8
ON595868	2018	Australia	HC-7
ON595867	2018	Australia	HC-6
ON595862	2018	Australia	HC-1
ON595866	2018	Australia	HC-5
MT239440	Sep-17	China	ZJU1709
MT239439	2016	China	ZJU1617
MW030110	May-18	China	SD
MW030109	May-18	United States	79-1146_CA
MW030108	May-18	China	QS
MT444152	25-May-19	China	HF1902
MN165107	Apr-18	China	XXN
KU215428	Jul-05	Belgium	Cat3_day28_withoutdeletion
KU215427	2013	Belgium	Cat3_day28_deletion
KU215426	2013	Belgium	Cat2_day84
KU215425	2013	Belgium	Cat1_day28_withoutdeletion
KU215424	2013	Belgium	Cat1_day28_deletion
KU215423	2013	Belgium	Cat3_day9
KU215422	2013	Belgium	Cat2_day21_withoutdeletion
KU215421	2013	Belgium	Cat2_day21_deletion
KU215420	2013	Belgium	Cat1_day7
KU215419	2013	Belgium	Inoculum
KF530123	17-Aug-10	Netherlands	*Felis catus*/NLD/UU88/2010
KP143512	1-Jan-13	United Kingdom	26M
KP143511	1-Jan-13	United Kingdom	80F
KP143510	1-Jan-13	United Kingdom	67F
KP143509	1-Jan-13	United Kingdom	65F
KP143508	1-Jan-13	United Kingdom	28O
KP143507	1-Jan-13	United Kingdom	27C
JN634064	30-Aug-11	United States	WSU 79-1683
JQ408980	13-Jan-12	Hungary	DF-2 R3i
JN183883	12-Mar-10	Netherlands	UU54
JN183882	14-Jul-10	Netherlands	UU47
HQ392469	22-Jan-08	Netherlands	UU40
HQ012372	11-Sep-07	Netherlands	UU34
FJ938058	7-Jun-07	Netherlands	UU16
FJ938060	30-Mar-93	United States	UU2
HQ392472	22-Jan-08	Netherlands	UU30
HQ392471	23-Aug-07	Netherlands	UU20
HQ392470	23-Aug-07	Netherlands	UU19
HQ012371	25-Jan-08	Netherlands	UU31
HQ012370	3-Jan-08	Netherlands	UU24
HQ012369	11-Sep-07	Netherlands	UU21
HQ012368	24-Jul-07	Netherlands	UU18
HQ012367	17-Jul-07	Netherlands	UU17
GU553362	13-Sep-07	Netherlands	UU23
GU553361	13-Sep-07	Netherlands	UU22
FJ938062	24-May-07	Netherlands	UU9
FJ938059	5-Jun-07	Netherlands	UU10
FJ938057	13-Jul-07	Netherlands	UU15
FJ938056	7-Mar-07	Netherlands	UU5
FJ938055	24-May-07	Netherlands	UU8
FJ938054	2-Feb-07	Netherlands	UU4
FJ938053	25-Apr-07	Netherlands	UU7
FJ938052	5-Jun-07	Netherlands	UU11
FJ938051	28-Jan-02	United States	RM
MZ420153	15-Mar-17	Haiti	Z19
MW591993	2017	Malaysia	CCoV-HuPn-2018
KP981644	2005	Italy	CB/05
JN856008	1976	United States	A76
GQ477367	Nov-08	China	CCoV/NTU336/F/2008
MZ320954	Feb-20	China	CCoV/GD/2020/X9
MZ320953	Feb-20	China	CCoV/GD/2020/X8
OP179857	2019	Brazil	CCov/PK02/2019/BRA
OM950729	2020	China	GH4-2
OM950728	2020	China	GH8-2
OM451123	2021	China	HeB-G1
OM451122	2021	China	SD-F3
MT955604	2-Sep-20	India	PVNRTVU/2020-0001
MT906865	2020	United Kingdom	2020 7
MT906864	2020	United Kingdom	2020/15
KY063618	1-Jul-16	China	HLJ-073
KY063617	1-Jul-16	China	HLJ-072
KY063616	1-Jul-16	China	HLJ-071
KC175341	1978	United States	S378
KC175340	1978	United States	K378
KC175339	1971	Germany	171
JQ404410	11-Jan-12	United States	TN-449
JQ404409	11-Jan-12	United States	1-71
KP849472	2003	Italy	23 03
DQ811788	22-Jun-06	United States	Attenuated Purdue P115
DQ811785	22-Jun-06	United States	Virulent Miller M6
DQ811789	1952	United States	Virulent Purdue
DQ811786	1987	United States	Partially attenuated Miller M60
KX499468	22-Dec-15	China	TGEV AHHF
KX900411	5-Feb-14	United States	TGEV/USA/SouthDakota154/2014
KX900410	4-Feb-14	United States	TGEV/USA/Minnesota153/2014
KX900409	31-Jan-14	United States	TGEV/USA/Minnesota152/2014
KX900408	17-Jan-14	United States	TGEV/USA/Wisconsin151/2014
KX900407	1-Mar-13	United States	TGEV/USA/Minnesota150/2013
KX900406	28-Feb-13	United States	TGEV/USA/Illinois149/2013
KX900405	4-Jan-13	United States	TGEV/USA/Minnesota148/2013
KX900404	29-Nov-12	United States	TGEV/USA/Oklahoma147/2012
KX900403	9-Apr-08	United States	TGEV/USA/Illinois146/2008
KX900402	17-Apr-08	Mexico	TGEV/Mex/145/2008
KX900401	15-Apr-08	United States	TGEV/USA/Tennessee144/2008
KX900400	6-Mar-08	United States	TGEV/USA/Iowa143/2008
KX900399	16-Feb-07	United States	TGEV/USA/NorthCarolina142/2007
X900398	8-Feb-07	United States	TGEV/USA/Minnesota141/2007
KX900397	8-Feb-07	United States	TGEV/USA/NorthCarolina140/2007
KX900396	17-Nov-06	United States	TGEV/USA/Illinois139/2006
KX900395	8-Nov-06	United States	TGEV/USA/Minnesota138/2006
KX900394	1988	United States	TGEV/USA/HB/1988
KX900393	8-Dec-06	United States	TGEV/USA/Z/1986
NC_038861	8-Feb-00	Spain	PUR46-MAD
MZ322950	21-Apr-19	China	CH/GX/TGEV/2662/2019
KU729220	10-Feb-98	China	TH-98
KP202848	7-Mar-13	China	SHXB
KC962433	5-May-12	China	TGEV-HX
AJ271965	9-Mar-01	United States	Purdue
ON016092	13-Mar-22	China	CHN-SC-H
OK078898	1990	Denmark	PRCV-1/90-DK
ON859974	2021/2/10	China	HNSQ-2021
MW804449	Nov-17	China	CH8438
OM802899	4-Jul-19	China	SZ19
MZ368889	24-Jun-20	China	HB-1
MT576083	Mar-16	China	HQ2016
KX083668	2015	China	HE-1
DQ443743	10-Mar-06	China	SC-Y
DQ201447	8-Sep-05	China	TS
HQ462571	1-Nov-10	China	WH-1
FJ755618	1973	China	H16
EU074218	24-Aug-09	China	Attenuated H
KT696544	2012	China	JS2012
HM776941	2009	China	AYU
KX058075	2012	China	CN12

Abbreviations: CoVs, coronaviruses; FIPV, feline infectious peritonitis virus.

## Data Availability

The data that support the findings of this study are available from the corresponding author upon reasonable request.

## References

[1] Doki T., Yabe M., Takano T., Hohdatsu T. (2018). Differential Induction of Type I Interferon by Type I and Type II Feline Coronaviruses in Vitro. *Research in Veterinary Science*.

[2] de Cássio V. de Barros B., de Castro C. M. O., Pereira D. (2019). First Complete Genome Sequence of a Feline Alphacoronavirus 1 Strain from Brazil. *Microbiology Resource Announcements*.

[3] Neuman B. W., Buchmeier M. J. (2016). Advances in Virus Research. *Advances in Virus Research*.

[4] Cook S., Castillo D., Williams S., Haake C., Murphy B. (2022). Serotype I and II Feline Coronavirus Replication and Gene Expression Patterns of Feline Cells—Building a Better Understanding of Serotype I FIPV Biology. *Viruses*.

[5] Wang G., Hu G., Liang R. (2021). Establishment of Full-Length cDNA Clones and an Efficient Oral Infection Model for Feline Coronavirus in Cats. *Journal of Virology*.

[6] Shi J., Wen Z., Zhong G. (2020). Susceptibility of Ferrets, Cats, Dogs, and Other Domesticated Animals to SARS-Coronavirus 2. *Science*.

[7] Tortorici M. A., Walls A. C., Joshi A. (2022). Structure, Receptor Recognition, and Antigenicity of the Human Coronavirus CCoV-HuPn-2018 Spike Glycoprotein. *Cell*.

[8] Cheng H.-Y., Jian S.-W., Liu D.-P. (2020). Contact Tracing Assessment of COVID-19 Transmission Dynamics in Taiwan and Risk at Different Exposure Periods Before and After Symptom Onset. *JAMA Internal Medicine*.

[9] Wang W., Deng Z., Jin Z. (2020). Bioinformatics Analysis and Prokaryotic Expression of FIPV AH1905 Strain N Gene. *Acta Agriculturae Zhejiangensis*.

[10] Drummond A. J., Suchard M. A., Xie D., Rambaut A. (2012). Bayesian Phylogenetics With BEAUti and the BEAST 1.7. *Molecular Biology and Evolution*.

[11] Kalyaanamoorthy S., Minh B. Q., Wong T. K. F., von Haeseler A., Jermiin L. S. (2017). ModelFinder: Fast Model Selection for Accurate Phylogenetic Estimates. *Nature Methods*.

[12] Turlewicz-Podbielska H., Augustyniak A., Pomorska-Mól M. (2022). Novel Porcine Circoviruses in View of Lessons Learned from Porcine Circovirus Type 2-Epidemiology and Threat to Pigs and Other Species. *Viruses*.

[13] Amoutzias G. D., Nikolaidis M., Tryfonopoulou E., Chlichlia K., Markoulatos P., Oliver S. G. (2022). The Remarkable Evolutionary Plasticity of Coronaviruses by Mutation and Recombination: Insights for the COVID-19 Pandemic and the Future Evolutionary Paths of SARS-CoV-2. *Viruses*.

[14] Wang Z., Li S., Shao Y. (2022). Genomic Characterization and Pathogenicity Analysis of a Porcine Deltacoronavirus Strain Isolated in Western China. *Archives of Virology*.

[15] Thayer V., Gogolski S., Felten S., Hartmann K., Kennedy M., Olah G. A. (2022). 2022 AAFP/EveryCat Feline Infectious Peritonitis Diagnosis Guidelines. *Journal of Feline Medicine and Surgery*.

[16] Osipiuk J., Azizi S.-A., Dvorkin S. (2021). Structure of Papain-Like Protease from SARS-CoV-2 and Its Complexes With Non-Covalent Inhibitors. *Nature Communications*.

[17] Shirato K., Chang H.-W., Rottier P. J. M. (2018). Differential Susceptibility of Macrophages to Serotype II Feline Coronaviruses Correlates With Differences in the Viral Spike Protein. *Virus Research*.

[18] Crawford A. H., Stoll A. L., Sanchez-Masian D. (2017). Clinicopathologic Features and Magnetic Resonance Imaging Findings in 24 Cats With Histopathologically Confirmed Neurologic Feline Infectious Peritonitis. *Journal of Veterinary Internal Medicine*.

[19] Yang H., Peng Q., Lang Y. (2022). Phylogeny, Evolution, and Transmission Dynamics of Canine and Feline Coronaviruses: A Retro-Prospective Study. *Frontiers in Microbiology*.

[20] Amer A., Suri A. S., Rahman O. A. (2012). Isolation and Molecular Characterization of Type I and Type II Feline Coronavirus in Malaysia. *Virology Journal*.

[21] Ibrahim O. M., Allawe A. B., Kadhim H. A. (2022). Isolation and Molecular Detection of Feline Infectious Peritonitis Virus. *Archives of Razi Institute*.

[22] Shin D., Mukherjee R., Grewe D. (2020). Papain-Like Protease Regulates SARS-CoV-2 Viral Spread and Innate Immunity. *Nature*.

[23] van Vliet V. J. E., Huynh N., Palà J. (2022). Ubiquitin Variants Potently Inhibit SARS-CoV-2 PLpro and Viral Replication via a Novel Site Distal to the Protease Active Site. *PLoS Pathogens*.

[24] Klaus J., Meli M. L., Willi B. (2021). Detection and Genome Sequencing of SARS-CoV-2 in a Domestic Cat With Respiratory Signs in Switzerland. *Viruses*.

[25] Zhao S., Li W., Schuurman N., van Kuppeveld F., Bosch B. J., Egberink H. (2019). Serological Screening for Coronavirus Infections in Cats. *Viruses*.

[26] Silva C. S., Mullis L. B., Pereira O. (2014). Human Respiratory Coronaviruses Detected In Patients With InfluenzaLike Illness in Arkansas, USA. *Virology & Mycology*.

[27] Kiss I., Poland A. M., Pedersen N. C. (2004). Disease Outcome and Cytokine Responses in Cats Immunized With An Avirulent Feline Infectious Peritonitis Virus (FIPV)-UCD1 and Challenge-Exposed With Virulent FIPV-UCD8. *Journal of Feline Medicine and Surgery*.

[28] Terada Y., Kuroda Y., Morikawa S. (2019). Establishment of a Virulent Full-Length cDNA Clone for Type I Feline Coronavirus Strain C3663. *Journal of Virology*.

