# Supplementary material for: Isolation and Genomic Characteristics of a Novel Pathogenicity Type I Feline Coronavirus in Mainland China
Source: Transbound Emerg Dis. 2024 Nov 13;2024:4162458. doi: 10.1155/2024/4162458 (PMC12016700; doi:10.1155/2024/4162458)
Supplement: Supporting Information — is available from the Wiley Online Library or from the author. [file 4162458.f1.docx]

**Table S1.** Primers used for FCV, FPV, FHVand FCoV PCR analysis

| Items | Sequences(5’-3’) | Amplification size | Annealing temperature |
| --- | --- | --- | --- |
| FPVVP2-F | 5‘-TGGTTCTGGGGGTGTGGG-3’ | 468 bp | 54℃ |
| FPVVP2-R | 5‘-GCTGCTGGAGTAAATGGC-3’ |  |  |
| FCoV-F | 5 '-TGCTATTAGTAAGTGGGGCC-3’ | 366bp | 52℃ |
| FCoV-R | 5 'CAACAACTTCCTAAACAACC3’ |  |  |
| FCVORF2-F | 5’-AACCTGCGCTAACGTGCTT-3’ | 922 bp | 54℃ |
| FCVORF2-R | 5’-CAGTGACAATACACCCAGAA-3’ |  |  |
| FHV-TK-F | :5’-GACGTGGTGAATTATCAG-3’ | 290 bp | 54℃ |
| FHV-TK-R | 5’-CAACTAGATTTCCACCAGG-3’ |  |  |
